# Emigration effects on estimates of age‐ and sex‐specific survival of two sciurids

**DOI:** 10.1002/ece3.8833

**Published:** 2022-04-23

**Authors:** Matthew J. Weldy, Damon B. Lesmeister, Clinton W. Epps

**Affiliations:** ^1^ Pacific Northwest Research Station U.S.D.A. Forest Service Corvallis Oregon USA; ^2^ 2694 Department of Fisheries, Wildlife, and Conservation Sciences Oregon State University Corvallis Oregon USA

**Keywords:** apparent survival, demography, emigration, *Glaucomys oregonensis*, immigration, *Neotamias townsendii*, site fidelity, vital rates

## Abstract

Age‐ and sex‐specific survival estimates are crucial to understanding important life history characteristics, and variation in these estimates can be a key driver of population dynamics. When estimating survival using Cormack–Jolly–Seber (CJS) models, emigration is typically unknown but confounded with apparent survival. Consequently, especially for populations or age classes with high dispersal rates, apparent survival estimates are often biased low and temporal patterns in survival might be masked when site fidelity varies temporally. We used 9 years of annual mark–recapture data to estimate age‐, sex‐, and time‐specific apparent survival of Humboldt's flying squirrels (*Glaucomys oregonensis*) and Townsend's chipmunks (*Neotamias townsendii*). For Humboldt's flying squirrels, these estimates support a small body of research investigating potential variation in survival among age and sex classes, but age‐ and sex‐specific survival has not been evaluated for Townsend's chipmunks. We also quantified the effects of age‐ and sex‐specific emigration on confounded estimates of apparent survival. Our estimates of juvenile flying squirrel survival were high relative to other small mammal species and estimates for both species were variable among years. We found survival differed moderately among age and sex classes for Humboldt's flying squirrels, but little among age and sex classes for Townsend's chipmunks, and that the degree to which emigration confounded apparent survival estimates varied substantially among years. Our results demonstrate that emigration can influence commonly used estimates of apparent survival. Unadjusted estimates confounded the interpretation of differences in survival between age and sex classes and masked potential temporal patterns in survival because the magnitude of adjustment varied among years. We conclude that apparent survival estimators are robust during some time periods; however, when emigration rates vary in time, the effects of emigration should be carefully considered and accounted for.

## Graphical abstract

Confounding between apparent survival and emigration is well established. However, adjusting apparent survival estimates with estimates of site fidelity separates the two temporal processes. This is important because site fidelity can be highly variable within and among species.

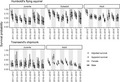

## 1 INTRODUCTION

Variation in survival rates can be a key driver of population dynamics (Cole, 1954), and thus is vital for the study of population demography and life history (Franklin et al., 1996). Within a population, the relative survival of juveniles and adults (Charlesworth, 1994) or males and females (Promislow, 1992) can help inform which factors are important to population dynamics and how populations change through time (Morrison & Hik, 2007). In mammals, adult and juvenile survival are often correlated, although juvenile survival is usually lower and more variable (Promislow & Harvey, 1990). This variation is thought to be regulated by juvenile life history characteristics such as natal dispersal (Rödel et al., 2015), or increased sensitivity to limited food (Jackson et al., 2001), thermoregulatory stress (Rödel et al., 2004), and predation (Garrett & Franklin, 1988). Similarly, there is a long‐standing belief that males exhibit lower survival rates than females (Vinogradov, 1998) because of exposure to higher costs of dispersal associated with locating and competing for mates (Promislow, 2003). Despite their importance in understanding the relative influence of survival on population dynamics, age‐, sex‐, and time‐specific estimates of survival are unavailable for many small mammal species because obtaining suitable data is challenging.

One common method for estimating survival is the Cormack–Jolly–Seber (CJS) model, which jointly estimates apparent survival and recapture probabilities (Cormack, 1964; Jolly, 1965; Seber, 1965). Apparent survival estimates from capture–recapture data and CJS models are commonly interpreted as estimates of survival; however, the estimated parameter is the product of true survival and site fidelity (Lebreton et al., 1992) because individual survival is indistinguishable from permanent emigration. If emigration is permanent or non‐random, CJS survival probability estimates will be biased low (Schaub et al., 2004) and the magnitude of bias can be large (Cooper et al., 2008; Horton & Letcher, 2008). A number of approaches have been suggested to deal with this bias. For example, a multistate approach allows for separation of movement and survival probabilities (Brownie et al., 1993), the robust design approach can account for temporary emigration (Pollock et al., 1990), and data integrations can allow joint estimation of true survival and site fidelity (Burnham, 1993). More recently, Gilroy et al. (2012) and Schaub and Royle (2014) developed CJS model extensions that adjusted estimates of apparent survival with those of site fidelity. In some cases, estimates of apparent survival will suffice for conservation or management. However, when little is known about species‐specific variation in survival (age or sex variation) or patterns of dispersal, inferences based on apparent survival estimates could mask important spatial or temporal variation in true survival.

Forest‐adapted small mammals are important to forest health as prey species and dispersal agents of hypogeous fungi and spermatophyte seeds (Trappe et al., 2009); yet, few studies have estimated movement rates (emigration, immigration, or site fidelity) for these species, and thus unbiased estimates of survival are rare or non‐existent. We focused our analyses on two small mammal species, Humboldt's flying squirrels (*Glaucomys oregonensis*; hereafter flying squirrel) and Townsend's chipmunks (*Neotamias townsendii*; hereafter chipmunk), which occur sympatrically in forested habitat throughout western Oregon. Both species are important components of the avian (including northern spotted owls *Strix occidentalis caurina*) and mammalian prey base (Forsman et al., 2001; Fryxell et al., 1999), and have broadly similar diets (Maser et al., 1978). Despite these similarities, these two species have different life history characteristics. Flying squirrels have been characterized as a potentially K‐selected species with “slow” traits (Bielby et al., 2007), leading to survival that is higher than for similar sized mammals (Smith, 2007; Villa et al., 1999) and that varies little across time (Lehmkuhl et al., 2006). However, other demographic characteristics such as abundance (Weldy et al., 2019), sex ratio (Rosenberg & Anthony, 1992), and recruitment (Weldy et al., 2020) vary substantially across time. Much less is known about chipmunk demography, but they have been characterized as an r‐selected species with “fast” traits (Bielby et al., 2007). For example, chipmunk population growth rates are primarily driven by recruitment, while survival is generally low and can exhibit substantial temporal variation (Weldy et al., 2020). Little is known about variation in survival among age or sex classes for either species, but the relative proportion of juvenile flying squirrels in northern spotted owl diets is seasonally variable (Forsman et al., 1994), which suggests potential for differences in juvenile mortality rates that could cause negative biases in apparent survival estimates.

Our objectives for this study were to estimate age‐, sex‐, and time‐specific annual survival and recapture probability for flying squirrels and chipmunks captured during a 9‐year period. Our study sites were located within old forests and during a period with little disturbance, and thus represent a different ecological context than small mammal studies in managed forests. We used mark–recapture data and two estimators to quantify sensitivity of survival estimates to variation in movement probabilities: (1) a CJS estimator that jointly estimates apparent survival and recapture probability, and (2) an integrated modeling approach to estimate immigration rates, which we used to derive site‐fidelity rates and emigration‐adjusted survival. Previous research in this system established that flying squirrels have higher survival rates than chipmunks (Weldy et al., 2020). Here, we hypothesized that survival would vary among age and sex classes and that differences in apparent survival among age and sex classes was confounded by variation in emigration rates. For juveniles of both species, we predicted lower survival probabilities and higher immigration rates relative to subadults and adults, but that adjusting apparent survival for emigration would reduce differences in survival among age classes (Dobson, 1982). We also predicted that males of both species would have lower survival and higher immigration rates relative to females because male mammals typically disperse more frequently and farther, and have higher predation risk and resource acquisition costs (Lemaître et al., 2020). Previous work by Weldy et al. (2020) estimating survival in this system was focused on links between abundance‐associated covariates and vital rates. In this study, we used an additional 3 years of data to estimate age‐, sex‐, and time‐specific variation in survival as well as the influence of temporal variation in immigration and emigration on the estimation of survival.

## 2 MATERIALS AND METHODS

### 2.1 Study area

**FIGURE 1:**
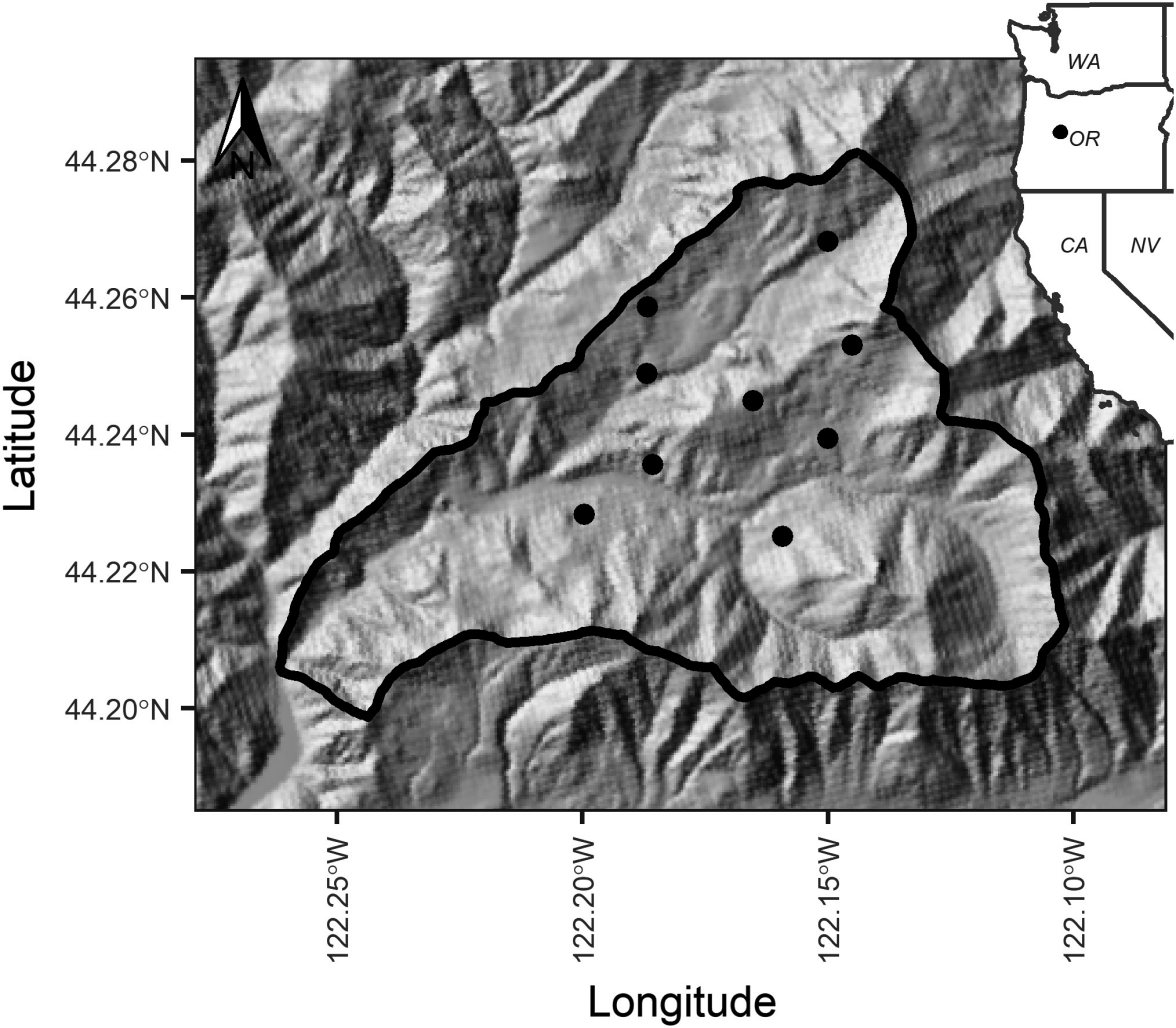
Location of nine live‐trapping arrays (dark points) within the H. J. Andrews Experimental Forest (black boundary) in Oregon where we sampled Humboldt's flying squirrels (*Glaucomys oregonensis*) and Townsend's chipmunks (*Neotamias townsendii*) during 2011–2019

We collected field data annually during September–November from 2011 to 2019 on nine sites in the H. J. Andrews Experimental Forest on the west slope of the Cascade Mountains in Oregon, United States (44°14’ N, 122°10’ W; Figure 1). The study sites were all located in a late‐successional forest (>400 years) dominated by large Douglas‐fir (*Pseudotsuga menziesii*), western hemlock (*Tsuga heterophylla*), and Pacific silver fir (*Abies amabilis*; Schulze & Lienkaemper, 2015). Understory characteristics on the study sites ranged from open understories to dense shrubs and common understory vegetation included black berry, raspberry, and salmonberry (*Rubus* spp.), common snowberry (*Symphoricarpos albus*), deer fern (*Blechnum spicant*), huckleberry (*Vaccinium* spp.), Oregon grape (*Mahonia aquifolium*), oxalis (*Oxalis* spp.), salal (*Gaultheria shallon*), sword fern (*Polystichum munitum*), and vine maple (*Acer circinatum*).

**FIGURE A1:**
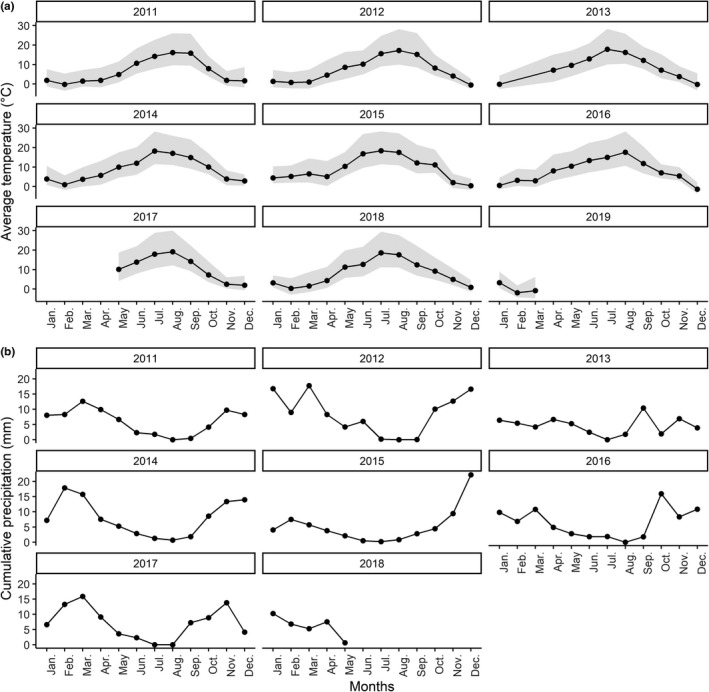
(a) Average monthly temperatures (°C; April 2011 to March 2019) and (b) cumulative monthly precipitation (mm; April 2011 to September 2018) recorded at the central meteorological (1020 m) weather station in the H. J. Andrews Experimental Forest. The grey color band around average monthly temperature values indicates the range (minimum to maximum) of temperatures recorded within the month

Weather on the study sites was typically warm and dry from May to September, and cool and wet from October to April when approximately 80% of the annual precipitation occurs. Annual precipitation primarily consists of rain at elevations <1000 m and snow at higher elevations (Bierlmaler & McKee, 1989). At 605 m, 30‐year (1981–2010) averages were 1955 mm of precipitation, 4.3°C minimum temperature, and 15.6°C maximum temperature (PRISM Climate Group, 2004). Monthly temperature and precipitation varied seasonally and inter‐annually during the period of our study (Figure A1).

### 2.2 Data collection

The nine study sites (7.84 ha each) were randomly selected from potential sites defined by three strata gradients of elevation (range = 683–1244 m) and canopy openness (range = 0–40%). The average distance among sites was 2963 m (range = 1078–5940 m). As described by Weldy et al. (2020), at each site, we established and conducted live trapping at 64 stations, each with two traps arranged in an 8 × 8 array with 40 m between stations. We conducted live trapping annually on each site during September, October, and November from 2011 to 2019. From 2011 to 2016, each site was trapped for 3 consecutive trapping weeks. During each trapping week, traps were checked once per day for four consecutive trap nights. Starting in 2017, we reduced trapping effort on all sites to 2 consecutive trapping weeks, and during 2019 the number of sites was reduced to three (Table A1). For flying squirrels, we assigned individual age to one of three classes using body mass, pelage, and reproductive measures outlined by Villa et al. (1999), which reliably differentiate juvenile, subadult, and adult flying squirrels. For chipmunks, we assigned individual age to one of two classes using body mass. Adult chipmunks were defined as heavier than 68 g for males and 67.5 g for females (Gashwiler, 1976). We were unable to differentiate subadult chipmunks from juvenile and adult chipmunks using a weight threshold. Individual age classes were validated for logical consistency so individual age classifications could only increase. Live‐trapping protocols were approved by the Oregon State University's Institutional Animal Care and Use Committee (ACUP #4191 2011–2013; #4590 2014–2016; #4959 2017–2019) and were consistent with the American Society of Mammalogists guidelines for the use of wild mammals in research and education (Sikes, 2016).

### 2.3 Analytical methods

We estimated apparent annual survival (*φ*) and recapture probability (*p*) for flying squirrels and chipmunks using mark–recapture data and CJS models (Cormack, 1964; Jolly, 1965; Seber, 1965) in a state–space formulation (Gimenez et al., 2007; Royle, 2008), with an additional submodel to estimate immigration rates (*I*). This hierarchical model consists of two state processes and one observation process. The first state process, *φ* (the probability of surviving and remaining in the study area), was linked with the observation processes *p* (the capture probability of a marked individual; Lebreton et al., 1992). The second state process, *I*, was independent from the *φ* process and the *p* process and was used to derive site‐fidelity rates (*r*) and emigration‐adjusted survival estimates (*φ*_adjusted_).

For the *φ* process, we first defined a latent variable *z_i_*_,_ *_t_* as the true state of individual *i* at time *t*, where a value of 1 indicated *i* was alive at *t* and a value of 0 indicated *i* was dead at *t*. We also defined a vector ***f***, where *f_i_* was the first capture occasion for individual *i*. We modeled the probability that *i* was alive at *t*+1, conditional on first capture, and being alive at *t*, as a Bernoulli trial where the success probability is the product of *φ_a_*_,_ *_s_*_,_ *_t_* and *z_i_*_,_ *_t_*.

$$z_{i,f_{i}}=1$$

$$z_{i,t+1}|z_{i,t}∼\text{Bernoulli}\left(z_{i,t}*\varphi_{a,s,t}\right).$$

We modeled variation in *φ* as a logit linear function of an age‐specific (*a*) intercept for *i* at *t*, where ages ranged 1–3 (juvenile, subadult, and adult) for flying squirrels and 1–2 (juvenile and adult) for chipmunks, and an additive age‐ and sex‐specific (*s*) effect, where the effect of sex differed by age, the sex variable was defined as 0 for males and 1 for females, and we included a zero‐centered age‐, sex‐, and time‐specific random effect with standard deviation *σ*_a,s,t_.

$$\text{log}\text{it}\left(\varphi_{a,s,t}\right)=\beta_{{\text{age}}_{i}}+\beta_{\text{sex}}*{\text{Sex}}_{i}+\epsilon_{a_{i},s_{i},t}$$

$$\epsilon_{a,s,t}∼\text{Normal}\left(0,\sigma_{a,s,t}^{2}\right).$$

We used a standard observation process for capture–recapture data *y_i_*_,_ *_t_*, where recaptures on each occasion from the second to the last trapping occasion were modeled as Bernoulli trials with success probability *p_i_*_,_ *_t_*. To determine the most supported model structure for the observation process, we considered nine logit linear model structures (seven univariate and two bivariate) for both species to account for variation in *p* (Table 1).

$$y_{i,t}|z_{i,t}∼\text{Bernoulli}\left(z_{i,t}*p_{i,t}\right).$$

**TABLE 1:** Description of variables considered in Cormack–Jolly–Seber models of recapture probability (*p*) for Humboldt's flying squirrels (*Glaucomys oregonensis*) and Townsend's chipmunks (*Neotamias townsendii*) fitted using mark–recapture data, 2011–2019, recorded in the H. J. Andrews Experimental Forest, near Blue River, Oregon

Model^a^	Description
Null	Constant effect
s	Site‐specific variation
b	Permanent behavioral effect indicating captures after first capture
t	A year‐specific fixed effect for each trapping occasion from 2011 to 2019
T	Trend from the first to the last trapping occasion 2011 to 2019
tRE	Temporal effects 2011–2019 treated as a normally distributed random effect with a mean of 0 and standard deviation *σ_t_*
mH	Individual‐level normally distributed random effect with a mean of 0 and standard deviation *σ_mH_*
b + tRE	Additive model including a behavioral effect and normally distributed random effect with a mean of 0 and standard deviation *σ_t_*
b + mH	Additive model including a behavioral effect and normally distributed random effect with a mean of 0 and standard deviation *σ_mH_*

^a^ Model structure for apparent annual survival for both species was held to the model structure of primary research interest: age‐, sex‐, and time‐specific apparent survival.

We used Poisson regression to estimate age‐, sex, and time‐specific immigration rates (*I_a_*_,_ *_s_*_,_ *_t_*) during *t* from *t* = 2 to the number of occasions. The response variable (Imm*_a_*_,_ *_s_*_,_ *_t_*) was the age‐, sex‐, and time‐specific counts of captured unmarked individuals.

$${\text{Imm}}_{a,s,t}∼\text{Poisson}\left(C_{a,s,t}*I_{a,s,t}\right).$$

We modeled variation in *I_a_*_,_ *_s_*_,_ *_t_* as a log‐linear function of an age‐specific effect, a sex‐specific effect, and a zero‐centered normally distributed time‐specific random effect with standard deviation *σ_t_*.

$$\log\left(I_{a,s,t}\right)=\beta_{\text{age}}+\beta_{\text{sex}}+\alpha_{t}$$

$$\alpha_{t}∼\text{Normal}\left(0,\sigma_{t}^{2}\right).$$

We assumed that age‐ and sex‐specific emigration at *t* was equal to *I_a_*_,_ *_s_*_,_ *_t_*_+_ *_1_*, and that age‐ and sex‐specific *r_a_*_,_ *_s_*_,_ *_t_* was the complement to emigration (i.e., *r_a_*_,_ *_s_*_,_ *_t_* = 1–emigration*_a_*_,_ *_s_*_,_ *_t_*). These assumptions were reasonable on the study sites, which were randomly placed within a large, continuous, old late‐successional forest where site edges did not reflect biological edges. We then derived estimates of emigration‐adjusted survival (*φ*_adjusted_), defined as:

$$\varphi_{\text{adjusted}\;a,s,t}=\frac{\varphi_{a,s,t}}{r_{a,s,t}}.$$

For the observation process, we used the Watanabe–Akaike information criterion (WAIC; Watanabe, 2010) to select the most supported model structure for *p* (Hooten & Hobbs, 2015; Vehtari et al., 2017). We considered the model with the smallest WAIC value and highest model support weight (*ω*), the most supported model. We used the relative change in WAIC (ΔWAIC) to evaluate models relative to the top‐ranking model, and because the estimate of WAIC is sensitive to the sample distribution, we estimated the 95% credible interval (CI) for the difference in WAIC from the top‐ranking model. We assessed the meaningfulness of a difference between two models based on the degree to which the CI for the difference did or did not overlap zero.

We evaluated goodness of fit for the CJS model using a posterior predictive check approach (Gelman et al., 2013) to estimate a Bayesian p‐value (Meng, 1994). The data are binary and standard fit statistics are uninformative about model fit. Thus, a Bayesian *p*‐value was derived as the proportion of times that Chi‐squared test statistics (Pearson, 1900) calculated for simulated datasets were higher than Chi‐squared test statistics for an aggregation of the observed datasets (i.e., individual row sums; Royle et al., 2014). Perfect agreement between the observed and simulated datasets occurs when the Bayesian *p*‐value equals 0.5.

We conducted all analyses using R version 3.6.1 (R Core Team, 2020). The models were fitted using JAGS software version 4.3.0 (Plummer, 2003) through the R2jags package version 0.6‐1 (Su & Yajima, 2020). We used diffuse priors for all parameters and evaluated prior sensitivity using two sets of priors. During model selection steps, each model was estimated with three independent chains of 5000 iterations following a burn‐in period of 2000 iterations. For inference, the top‐ranking models for flying squirrels and chipmunks were estimated with three independent 50,000 iteration chains each following a burn‐in period of 50,000 iterations. We assessed model convergence by visual examination of trace plots and computed the Brooks–Gelman–Rubin convergence diagnostic ($\hat{R}$; Brooks & Gelman, 1998). We described the posterior distributions for each parameter by their mean and CI and assessed the strength of individual effects or the magnitude of difference between estimates based on the degree to which the CI for the estimate did or did not overlap zero.

## 3 RESULTS

We live trapped 117,432 trap nights and captured 1403 individual flying squirrels (692 females and 711 males) and 4394 individual chipmunks (1825 females and 2569 males). Of these, we estimated *φ* for the 1272 flying squirrels and 3873 chipmunks that were captured before the final trapping occasion. Average individual captures were 28.7 (range = 4–57 per year at each site) flying squirrels per year at each site and 69.1 (range = 19–165 per year at each site) chipmunks per year at each site. Site‐ and year‐specific (2012–2019) counts of unmarked individuals ranged 0–12 for flying squirrels and 3–54 for chipmunks.

**FIGURE 2:**
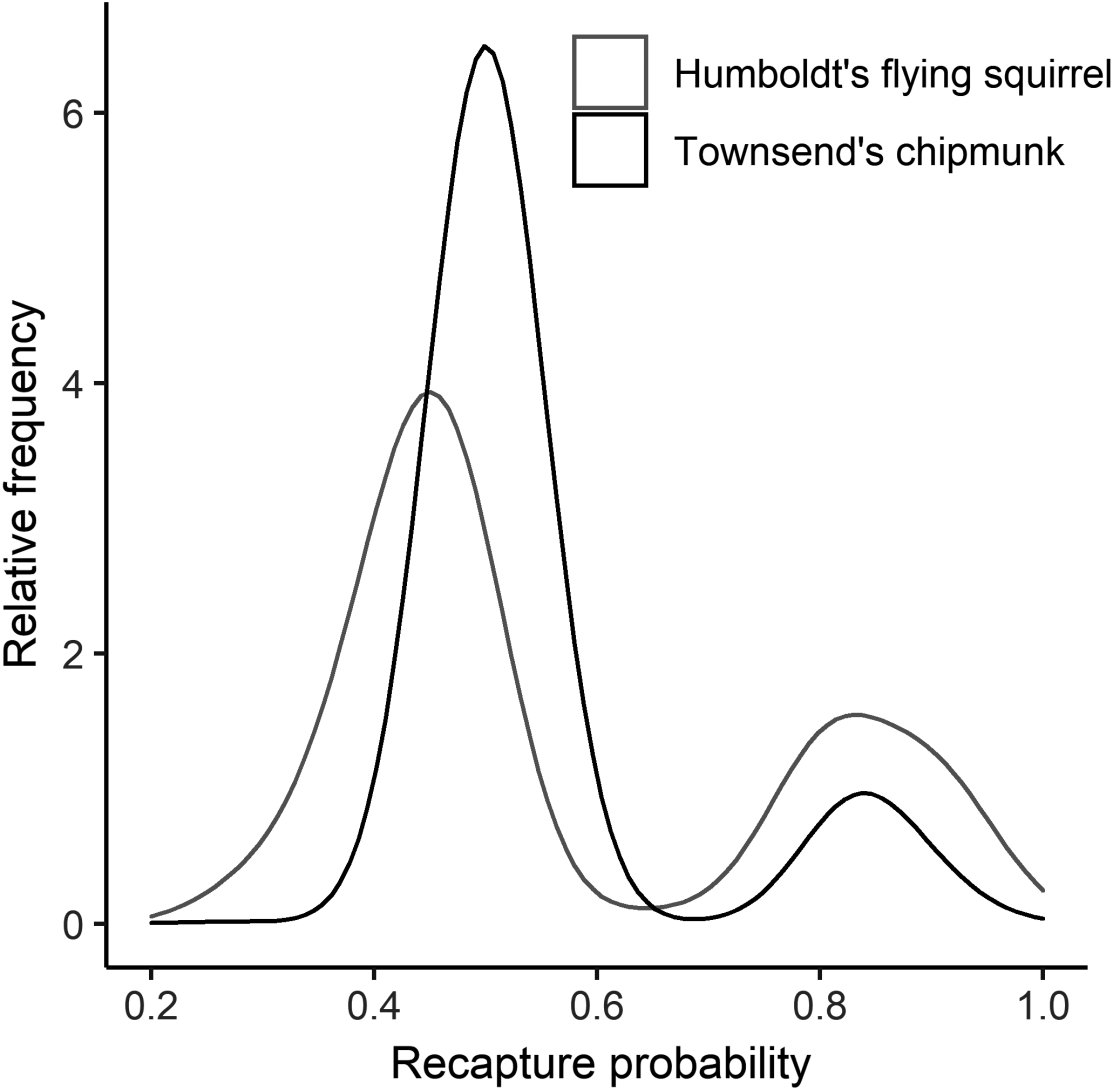
Relative frequencies of individual recapture probabilities for Humboldt's flying squirrels (*Glaucomys oregonensis*) and Townsend's chipmunks (*Neotamias townsendii*) estimated from models including an individual‐level random effect describing the observation process. The bimodal density plots are displayed with a Gaussian kernel using a smoothing bandwidth of 0.05

The most supported model of *p* for flying squirrel included the additive effects of a mean intercept and an individual‐level random effect (Table 2; Table A2). Mean *p* of flying squirrels was 0.66 (CI: 0.51–0.73), but individual estimates varied substantially (*σ_mH_* = 4.26, CI: 3.19–4.96), ranged 0.25 (CI: 0.02–0.69) to 0.96 (CI: 0.76–0.99), and had a bi‐modal posterior density with a dominant peak at approximately 0.45 and a smaller secondary peak at 0.81 (Figure 2). The most supported model of *p* for chipmunk included the additive effects of a mean intercept, a permanent behavioral trap response, and an individual‐level random effect (Table 2; Table A2). Mean *p* of chipmunks was 0.37 (CI: 0.01–0.94), and the probability of recapture of individuals after encountering traps was 0.77 (CI: 0.74–0.80). Individual *p* of chipmunks varied substantially (*σ_mH_* = 4.19, CI: 2.9–4.96), ranged 0.21 (CI: 0.01–0.62) to 0.93 (CI: 0.61–0.99), and had a bi‐modal posterior density with a dominant peak at approximately 0.50 and a much smaller secondary peak at 0.83 (Figure 2).

**TABLE 2:** Top three ranking models used to estimate recapture probability (*p*) of Humboldt's flying squirrels (*Glaucomys oregonensis*; HFS) and Townsend's chipmunks (*Neotamias townsendii*; TC) on nine late‐successional forest sites in the H. J. Andrews Experimental Forest, 2011–2019

Species	Model^a^	ΔWAIC	ΔWAIC 95% CI		*ω*
			Lower	Upper	
HFS	mH	0	NA	NA	1
	s	32	12.195	51.805	0
	b + tRE	48.43	26.907	69.953	0
	Null	469.83	451.498	488.162	0
TC	b + mH	0	NA	NA	1
	mH	91.24	71.168	111.312	0
	b + tRE	449.28	428.737	469.823	0
	Null	658.99	640.594	677.386	0

Note

Column headings indicate the species, recapture probability model structure, the relative change in Watanabe–Akaike information criterion (ΔWAIC) from the top‐ranking model, the lower 95% credible level (CI) for the relative change (lower), the upper CI interval for the relative change (upper), and the model support weight (*ω*).

^a^ Model structures are defined in Table 1.

Immigration rates varied among years for flying squirrels (*σ_t_* = 2.88, CI: 1.76–4.59), but less so for chipmunks (*σ_t_* = 1.41, CI: 0.83–2.55), and the species‐specific pattern of temporal variation was similar for both sexes. Flying squirrel immigration rates for females and males were low (≤0.11) for both age classes during six of eight study occasions (2013–2016, 2018, 2019), but were much higher (~0.22) for females and males of both age classes in 2012 and 2017 (Table A3). Similarly, chipmunk immigration rates were relatively low during six of eight study occasions (2012, 2014–2018), and were much higher for females and males during two occasions (2013, 2019; Table A3). Chipmunk immigration rates were higher relative to flying squirrel immigration rates during all occasions. We observed weak evidence that immigration rates of subadult and adult flying squirrels were lower than for juveniles, with <10% of the coefficient CI overlapping zero (*β*_Age_ = −0.13, CI: −0.28–0.02). Female flying squirrels (*β*_Sex_ = −0.21, CI: −0.36–−0.06) and chipmunks (*β*_Sex_ = −0.33, CI: −0.41–−0.25) had lower immigration rates than males of those species, respectively.

**FIGURE 3:**
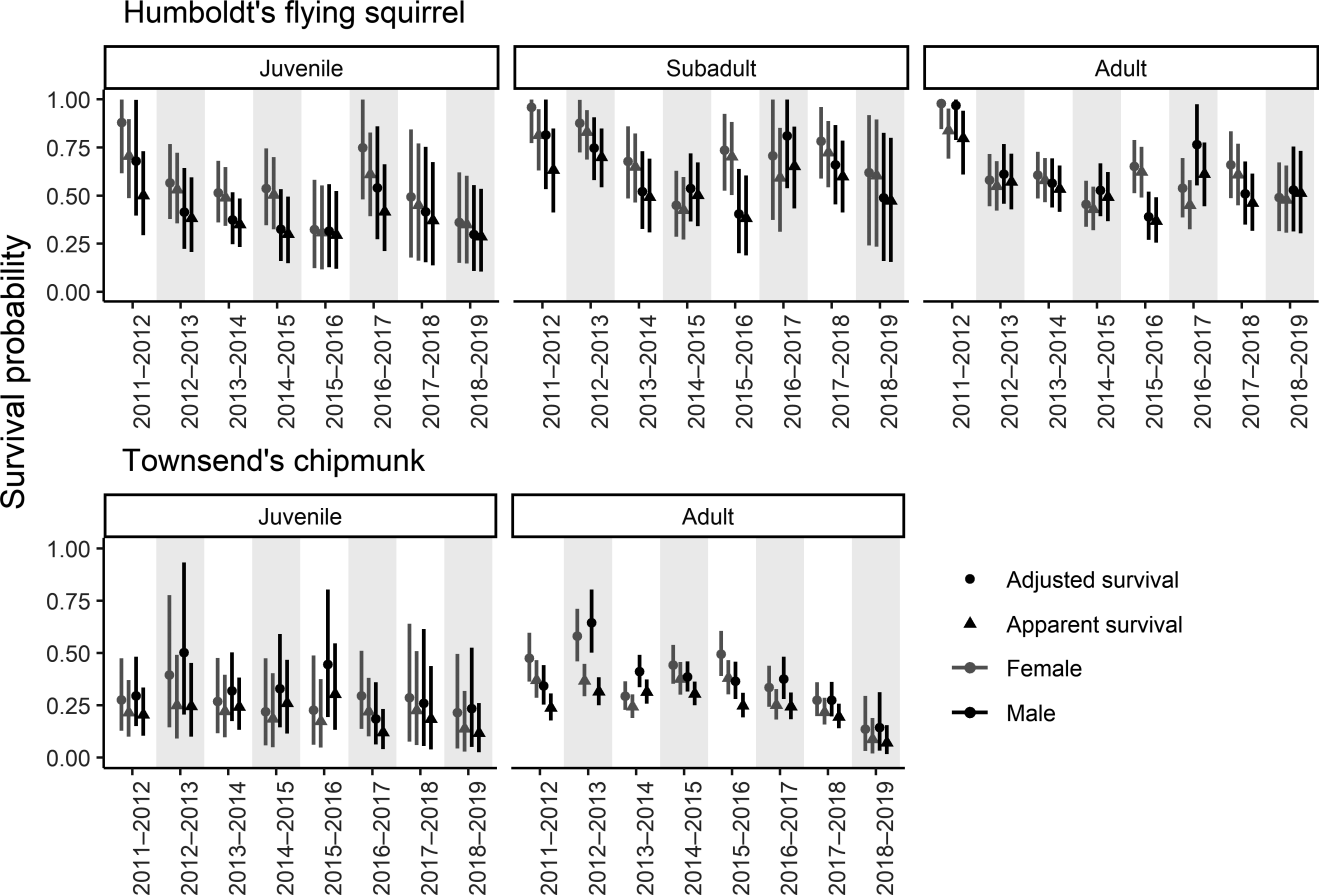
Estimates (mean and 95% credible interval) of annual apparent survival (*φ*, triangles) and adjusted survival (*φ*_adjusted_, circles) for female (grey) and male (black) Humboldt's flying squirrels (*Glaucomys oregonensis*) and Townsend's chipmunks (*Neotamias townsendii*), 2011–2019, in the H. J. Andrews Experimental Forest in Oregon

**FIGURE 4:**
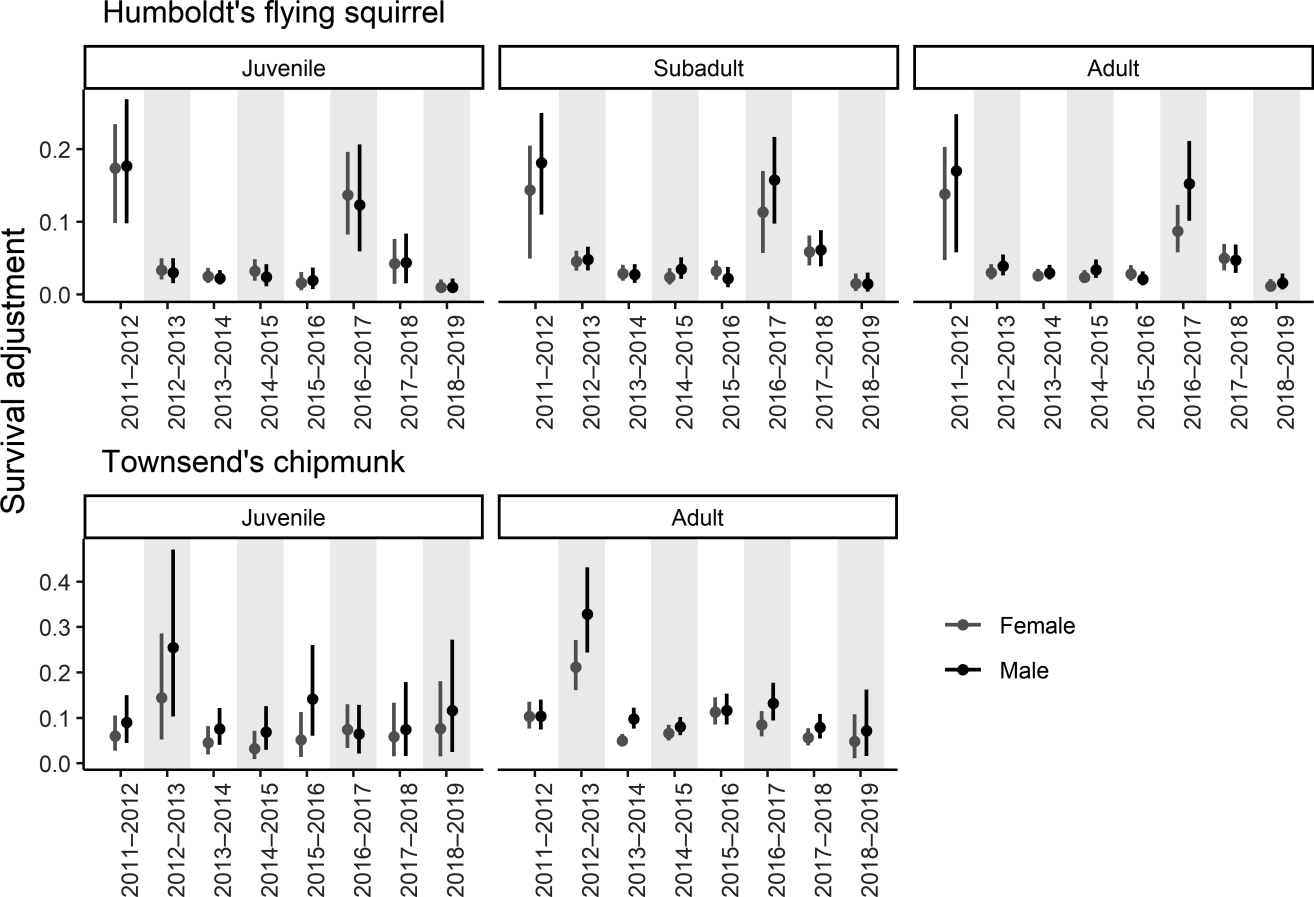
Age‐ and sex‐specific apparent survival emigration adjustment ($\Delta_{\text{survival adjustment}}=\varphi_{\text{adjusted}}‐\varphi$) estimates (mean and 95% credible intervals) for Humboldt's flying squirrels (*Glaucomys oregonensis*) and Townsend's chipmunks (*Neotamias townsendii*) captured 2011–2019 on the H. J. Andrews Experimental Forest in Oregon

For flying squirrels, *φ* estimates varied among age, year, and sex (*σ_a_*_,_ *_s_*_,_ *_t_* = 0.73, CI: 0.46–1.06). For female flying squirrels, *φ* ranged 0.31 (CI: 0.12–0.55) to 0.71 (CI: 0.49–0.90) for juveniles, 0.43 (CI: 0.27–0.60) to 0.83 (CI: 0.69–0.94) for subadults, and 0.43 (CI: 0.32–0.55) to 0.84 (CI: 0.69–0.95) for adults (Figure 3). For male flying squirrels, *φ* ranged 0.29 (CI: 0.11–0.53) to 0.50 (CI: 0.29–0.73) for juveniles, 0.39 (CI: 0.19–0.61) to 0.70 (CI: 0.54–0.85) for subadults, and 0.37 (CI: 0.26–0.49) to 0.80 (CI: 0.61–0.94) for adults. Pairwise differences in *φ* and *φ*_adjusted_ among flying squirrel age and sex classes were generally small and the CIs for the pairwise differences broadly overlapped zero. We found weak evidence that female juvenile *φ* was lower relative to female subadult *φ* ($\varphi_{\text{female}\;\text{juvenile}}‐\varphi_{\text{female}\;\text{subadult}}=‐0.19$, CI: −0.41–0.03) and male juvenile *φ* was lower relative to both male subadult *φ* ($\varphi_{\text{male}\;\text{juvenile}}‐\varphi_{\text{male}\;\text{subadult}}=‐0.20$, CI: −0.40–0.02) and male adult *φ* ($\varphi_{\text{male}\;\text{juvenile}}‐\varphi_{\text{male}\;\text{adult}}=‐0.18$, CI: −0.37–0.02). The rank order of *φ* among age classes was more variable among years for female flying squirrel relative to males, with all age classes represented as the minimum and maximum of the within‐year estimates at least once during 8 years (Figure 3). For male flying squirrels, juvenile *φ* was lowest among within‐year estimates during all 8 years, with the maximum of within‐year estimates alternating between the subadult (maximum 5 of 8 years) and adult (maximum 3 of 8 years) age classes (Figure 3). Emigration adjustments varied temporally, ranged 0.01 (CI: 0.00–0.02) to 0.18 (CI: 0.10–0.27), and were substantial during the 2011–2012 and 2016–2017 intervals (Figure 4). The magnitude of differences in adjustments among age and sex classes was small (Table A4).

For chipmunks, annual *φ* varied among years, but differed much less among age and sex relative to flying squirrels (*σ_a_*_,_ *_s_*_,_ *_t_* = 0.69, CI: 0.39–1.13). Annual *φ* ranged 0.13 (CI: 0.03–0.32) to 0.25 (CI: 0.09–0.50) for juvenile females and ranged 0.09 (CI: 0.02–0.19) to 0.38 (CI: 0.30–0.46) for adult females (Figure 3). For males, annual *φ* ranged 0.11 (CI: 0.03–0.26) to 0.30 (CI: 0.13–0.55) for juveniles and 0.07 (CI: 0.02–0.16) to 0.31 (CI: 0.26–0.37) for adults (Figure 3). Pairwise differences in *φ* and *φ*_adjusted_ among each chipmunk sex and age class were small and the CI for each difference broadly overlapped zero. For both sexes, the rank order of juvenile *φ* was lower than adult survival during most years (female: 7 of 8 years, male: 6 of 8 years). Emigration adjustments ranged 0.03 (CI: 0.01–0.07) to 0.33 (CI: 0.24–0.43), varied temporally for both sexes, and were substantial during the 2012–2013 interval (Figure 4). Emigration adjustments were larger in magnitude for males relative to females during all years (Table A4).

**FIGURE A2:**
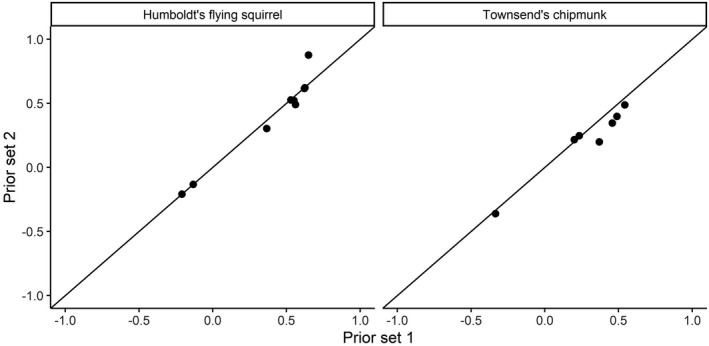
Prior sensitivity plot for uninformative prior sets 1 and 2 used to estimate apparent annual survival, recapture probability, and immigration rates of Humboldt's flying squirrels (*Glaucomys oregonensis*) and Townsend's chipmunks (*Neotamias townsendii*) captured during 2011–2019 in the H. J. Andrews Experimental Forest in Oregon

Covariate posterior distributions were similar for both sets of priors (Table 3, Figure A2). Visual inspection of trace plots and estimates of the Brooks–Gelman–Rubin convergence diagnostic indicated convergence was obtained for all monitored parameter estimates ($\hat{R}$ <1.03). Bayesian *p*‐values estimated from the posterior predictive checks were 0.53 for flying squirrels and 0.44 for chipmunks, indicating adequate fit for all models and suggesting that both candidate models generated data consistent with the observed data.

**TABLE 3:** Time‐invariant age‐ and sex‐specific estimates of mean apparent annual survival (*φ*_;_ real scale) and associated 95% credible intervals (CI, lower: 2.5%, upper: 97.5%) for Humboldt's flying squirrels (*Glaucomys oregonensis*; HFS) and Townsend's chipmunks (*Neotamias townsendii*; TC) captured 2011–2019 on the H. J. Andrews Experimental Forest in Oregon

Species	Age	Sex	Prior 1			Prior 2		
			Mean	95% CI		Mean	95% CI	
				Lower	Upper		Lower	Upper
HFS	J	M	0.366	0.232	0.518	0.301	0.190	0.438
	SA	M	0.561	0.404	0.709	0.488	0.350	0.637
	A	M	0.550	0.418	0.685	0.518	0.400	0.645
	J	F	0.622	0.404	0.805	0.618	0.429	0.786
	SA	F	0.625	0.407	0.813	0.621	0.432	0.788
	A	F	0.530	0.344	0.711	0.532	0.365	0.694
TC	J	M	0.203	0.110	0.325	0.257	0.135	0.416
	A	M	0.239	0.154	0.344	0.281	0.176	0.403
	J	F	0.486	0.261	0.711	0.494	0.252	0.737
	A	F	0.545	0.361	0.712	0.548	0.346	0.733

Note

Super columns “Prior 1” and “Prior 2” refer to estimates obtained using two covariate prior sets, and strong differences would indicate model sensitivity to prior selection. Age and sex categories include juvenile (J), subadult (SA), adult (A), male (M), and female (F).

## 4 DISCUSSION

Our analysis reinforces the need to adjust for emigration in estimates of apparent survival and demonstrates that reducing biases associated with emigration can result in much higher survival estimates than expected. We also present an analytical method to parse confounded survival and movement probabilities to reduce bias in empirical apparent annual survival estimates relative to emigration‐adjusted survival. After reducing bias associated with emigration, we revealed that juvenile flying squirrel survival may be much higher than anticipated based on previous studies, as our estimates are among the highest observed for any small mammal species (Table A5; Kraus et al., 2005). Indeed, our emigration‐adjusted survival for juvenile flying squirrels exceeded reported adult apparent annual survival estimates for many other species (Schaub & Vaterlaus‐Schlegel, 2001), including adult chipmunks in our study. Observing such high survival for juveniles of a small mammal species, even one described as having a relatively “slow” life history, is noteworthy because small‐bodied mammals typically have low survival rates (Blueweiss et al., 1978). Given that juveniles are expected to be more vulnerable to competition and predation (Dickman et al., 1991; Garrett & Franklin, 1988; Hill et al., 2019; Koivunen et al., 1996; Sakai & Noon, 1997 *)*, particularly for species with high adult survival, our finding suggests that resource availability was high during portions of the study period. Our approach also uncovered temporal variation in apparent survival biases that are likely present in a wide range of species and ecosystems.

Our findings were consistent with previous studies that demonstrated sometimes substantial negative biases of apparent survival relative to true survival caused by confounding of survival and emigration probabilities (Lebreton et al., 1992; Schaub & Royle, 2014). Beyond that widely recognized phenomenon (Schaub et al., 2004; Schaub & Royle, 2014), we demonstrated that levels of bias in apparent survival can vary substantially over time due to temporal variation in site‐fidelity rates. For both species examined here, the bias induced by confounding of emigration and survival probabilities was not consistent among years. In general, we observed consistent agreement between apparent survival and adjusted survival estimates for both species, sexes, and all age classes. However, during some years, the bias for one or more age‐ and sex‐specific classes was >1 order of magnitude larger than the bias in other years, and during those years, inferences for temporal variation in apparent survival and adjusted survival differed. For example, our estimates of apparent annual survival for juvenile and subadult male flying squirrels during 2016–2017 and 2017–2018 are similar, whereas mean emigration‐adjusted survival is much higher during 2016–2017 relative to 2017–2018. This is concerning for unadjusted apparent survival estimates, especially for short‐term studies that cannot differentiate between years when apparent survival is a suitable estimator for survival and years when movement probabilities are important and influential confounders. Moreover, differences in survival probabilities or movement behavior among age or sex classes could further confound estimation of either quantity individually (Schaub & Royle, 2014).

For flying squirrels, our estimates of adult apparent annual survival and adjusted survival were intermediate to previously reported estimates that ranged 0.32–0.68 (Gomez et al., 2005; Lehmkuhl et al., 2006; Ransome & Sullivan, 2002), including those reported by Weldy et al. (2020) for data collected on these sites during 2011–2016. In that analysis, temporal variation in apparent annual survival was not supported by model selection criteria, consistent with the findings of Lehmkuhl et al. (2006). But, in this analysis, we focused on estimates of age‐ and sex‐specific survival and estimated substantial temporal variation in apparent annual survival, especially for the juvenile and subadult age classes, suggesting additional years of data collection were necessary to adequately characterize temporal variance in survival (Hilde et al., 2020). In the expanded time series of mark–recapture data used here, our estimates of apparent annual survival during 2012–2016 were similar to those reported by Weldy et al. (2020) and showed little temporal variation, especially for the adult age class. However, during 2016–2019, we observed a peak and subsequent decline in apparent survival and emigration‐adjusted survival. For chipmunks, our estimates of apparent annual survival and adjusted survival were similar to conspecifics and congenerics (Schulte‐Hostedde et al., 2002; Weldy et al., 2020). However, we found less evidence for temporal variation in apparent annual survival, and temporal variation in adjusted survival did not match previously reported patterns (Weldy et al., 2020). Consistent with our hypotheses, juvenile flying squirrel survival was lower relative to subadults or adults, and male survival was lower relative to females for all three age classes, but the magnitude of that difference diminished with age. We also expected lower survival in juvenile chipmunks, but we found little evidence for a difference in age‐ or sex‐specific survival. For both species, however, temporal variation in survival within age and sex classes was larger than variation among age and sex classes, highlighting the importance of long‐term studies to understand variation in demographic traits (Doak et al., 2005).

On these old and relatively undisturbed forested sites, we expect that long‐term population persistence would reflect a balance in mortality and fecundity (Stearns, 1992; Sutherland et al., 1986). For flying squirrels, our findings suggest some evidence for an age‐structured population on our study sites, where adult mortality is likely compensated for by high juvenile survival rates. Previous studies suggest that the importance of age‐specific survival will vary regionally (Lehmkuhl et al., 2006; Villa et al., 1999), and identifying drivers of this variation is an important knowledge gap. For chipmunks, we found no evidence for age‐structured survival rates, and conclude that population persistence is more likely maintained through high reproductive effort. Reproductive lifespan is unlikely to compensate for adult chipmunk mortality because survival of adult chipmunks in these populations was low. Similarly, we suggest that age at first reproduction will not compensate for adult chipmunk mortality on these sites because the trait is unlikely to show temporal variation. Chipmunks are capable of reproduction in the spring (males) and summer (females) following their first winter (Gashwiler, 1976; Sullivan et al., 1983).

We found weak evidence that male survival was lower relative to female survival for flying squirrels and chipmunks, but for both species the effect size was small relative to temporal variation, and for flying squirrels, the difference observed in younger age classes did not persist for the adult age class. We are unclear what mechanism would cause an age‐structured pattern in the differences in survival between males and females, but we can suggest two hypothetical explanations. First, young female flying squirrels may delay natal dispersal until they are adults, or disperse smaller distances into more familiar nearby habitat relative to males. Both decisions might decrease predation risk for females relative to young dispersing males. Second, young male flying squirrels may undergo stronger competitive forces to secure habitat during natal dispersal or to find a mate during the subadult year (Bonduriansky et al., 2008; Carranza & Pérez‐Barbería, 2007; Vinogradov, 1998).

The importance of estimating age‐specific survival while correcting for emigration is demonstrated by contrasting findings of this study with those of previous research in this system. Weldy et al. (2020) observed a negative association between apparent annual survival and recruitment rate for chipmunks, where low apparent survival was coupled with high recruitment and low recruitment was coupled with high survival. If age‐specific survival was a primary driver of this observation, we expected to observe relatively stable survival of adult chipmunks while juvenile survival varied. Instead, we conclude that the low survival estimates coupled with high recruitment were associated with individual movement. Recruitment was large because individuals moved into study populations, while survival was low because marked individuals left those populations.

Immigration rates of flying squirrels were generally low, except in 2012 and 2017, when estimated immigration rates were more than twofold higher. In the intervals preceding these high immigration estimates, flying squirrel survival was high. This may have resulted in a surplus of individuals, which in turn prompted density‐driven emigration to find suitable habitat. The subsequent drops in survival after these high immigration estimates suggest that the outcome for many of these emigrating individuals was poor. For chipmunks, in comparison, immigration rates were more variable and much higher overall. For example, the lowest estimates of chipmunk immigration rates were nearly equivalent to the two peak flying squirrel immigration estimates. Taken together, these estimates demonstrate different temporal patterns of immigration, and consequently temporal variation in the influence of emigration on estimates of apparent survival. During most years, immigration is likely an unimportant driver of flying squirrel population dynamics, whereas it is likely consistently influential to chipmunk population dynamics. Little is known about the movement ecology of small mammals in the Pacific Northwest; however, these results suggest that further research exploring the drivers of temporal variation immigration rates could improve understanding of population dynamics. For example, we note that both years with the highest immigration rates (2012 and 2017) followed winters with high rainfall (Figure A1b). Future analyses should test the relative importance of climatic variables and intrinsic population processes as drivers of immigration rates.

We were concerned that age‐ or sex‐specific variation in site‐fidelity rates would bias inferences about differences in survival. We chose to use a submodel extension to the CJS model framework that estimated age‐, sex‐, and time‐specific immigration probabilities, which we used to derive estimates of site‐fidelity rates and adjusted survival. This approach is similar to others that integrate data to estimate adjusted survival (Abadi et al., 2010), which typically parameterize immigration as a Poisson‐distributed rate or count (Schaub & Fletcher, 2015). Early integrated analyses suggested that a crucial assumption of integrated models is that the datasets to be integrated are independent (Besbeas et al., 2002; Schaub & Abadi, 2011); violation of that assumption is thought to result in overestimates of precision (Lebreton et al., 1992). However, Weegman et al. (2020) found no effects on parameter bias or precision from integrated population models fit to simulated data with complete overlap.

Employing the emigration correction on survival estimates by our method requires carefully considering whether source or sink habitats exist within the study area. Our immigration submodel extension was contingent on the assumption that emigration rates at time *t* are equivalent to immigration rates at time *t* + 1 and that the site‐fidelity rates were complement to emigration rates. This is equivalent to the assumption that our study species are moving through the study system randomly in an even flow within a year. We felt that this assumption was met on our study sites, which were randomly placed within a large, continuous, old late‐successional forest where site edges did not reflect biological edges. Furthermore, Carey (1995) suggested that there was no evidence that the densities of flying squirrels and chipmunks were misleading indicators of habitat suitability, and Weldy et al. (2019) found no evidence for marginal or sink habitat on our study sites. Presence of sink habitats would have indicated that individuals were more likely to emigrate from or immigrate to specific sites. In that case, apparent annual survival estimates would be biased low, and violation of our random and even movement adjustment assumption would have caused our adjustment to apparent annual survival to overcompensate for the negative biases caused by movement on some sites.

Our study demonstrates a novel approach to gaining insight into links between movement and survival for two species of small mammals. The study of small mammal movement ecology often lags behind that of larger species, leading to knowledge gaps such as how movement of small mammals influences ecosystem health through the dispersal of hypogeous fungi spores and plant seeds (Trappe et al., 2009). However, while our methods are appropriate given the homogenous nature of our study area, the necessary assumptions might not hold in other systems with more fragmentation, variable habitat quality, or potential for source–sink dynamics among sites. Future studies could continue to explore the movement ecology of small mammals by incorporating study designs suitable for directly estimating movement parameters (i.e., multistate mark–recapture or telemetry) and evaluating the effects of spatiotemporal predictors on movement probabilities, or by linking temporal variation in movement rates to studies of population cycling (Fryxell et al., 1998; Weldy et al., 2019).

## AUTHOR CONTRIBUTIONS

**Matthew J. Weldy:** Conceptualization (equal); Data curation (equal); Formal analysis (lead); Investigation (equal); Methodology (equal); Visualization (equal); Writing – original draft (lead); Writing – review & editing (equal). **Damon B. Lesmeister:** Conceptualization (equal); Data curation (equal); Funding acquisition (equal); Investigation (equal); Project administration (equal); Supervision (equal); Validation (equal); Writing – review & editing (equal). **Clinton W. Epps:** Conceptualization (equal); Data curation (equal); Funding acquisition (equal); Investigation (equal); Project administration (equal); Supervision (equal); Validation (equal); Writing – review & editing (equal).

## Data Availability

Data used to estimate apparent annual survival and immigration for flying squirrels and chipmunks are available from figshare: https://figshare.com/projects/Small_Mammal_Age‐Specific_Survival/119784. R and JAGS code to recreate the analyses and figures is available on GitHub https://github.com/MJWeldy/SM_MAMM_AGE_SURVIVAL.

## APPENDIX 1

### “Emigration effects on estimates of age‐ and sex‐specific survival of small mammals” – Weldy et al.

**TABLE A1:** Site‐ and year‐specific trapping effort for Humboldt's flying squirrels (*Glaucomys oregonensis*; HFS) and Townsend's chipmunks (*Neotamias townsendii*; TC) on nine late‐successional forest sites in the H. J. Andrews Experimental Forest 2011–2019. Rows are indexed by the site number, columns 2–10 indicate trapping year, and values indicate the number of nights trapped at a site‐ and year‐specific occasion. A value of “NA” indicates that a grid was not trapped during that year

Site	Years								
	2011	2012	2013	2014	2015	2016	2017	2018	2019
1	12	12	12	12	12	12	8	8	NA
2	12	12	12	12	12	12	8	8	8
3	12	12	12	12	12	12	8	8	NA
4	12	12	12	12	12	12	4	8	NA
5	12	12	12	12	12	12	8	8	8
6	12	12	12	12	12	12	6	8	NA
7	12	12	12	12	12	12	8	8	NA
8	12	12	12	12	12	12	7	8	8
9	12	6	12	12	12	12	4	8	NA

**TABLE A2:** Model selection results used to estimate recapture probability (*p*) of Humboldt's flying squirrels (*Glaucomys oregonensis*; HFS) and Townsend's chipmunks (*Neotamias townsendii*; TC) on nine late‐successional forest sites in the H. J. Andrews Experimental Forest 2011–2019. Column headings indicate the species, recapture probability models structure, the relative change in Watanabe–Akaike information criterion (ΔWAIC) from the top‐ranking model, the lower 95% credible level (CI) for the relative change (Lower), the upper 95% credible level for the relative change (Upper), and the model support weight (*ω*)

Species	Model^a^	ΔWAIC	ΔWAIC 95% CI		*ω*
			Lower	Upper	
HFS	mH	0	NA	NA	1
	s	32.00	12.195	51.805	0
	b + tRE	48.43	26.907	69.953	0
	b + mH	55.53	33.985	77.075	0
	tRE	57.77	53.497	62.043	0
	T	108.49	89.870	127.110	0
	b	250.18	232.500	267.860	0
	t	413.72	395.333	432.107	0
	Null	469.83	451.498	488.162	0
TC	b + mH	0	NA	NA	0
	mH	91.24	71.168	111.312	0
	b + tRE	449.28	428.737	469.823	0
	b	509.36	490.014	528.706	0
	tRE	510.88	508.085	513.675	0
	s	592.63	575.126	610.134	0
	Null	658.99	640.594	677.386	0
	t	666.00	648.158	683.842	0
	T	683.62	666.198	701.042	0

^a^ Model structures are defined in Table 1.

**TABLE A3:** Age‐ and sex‐specific estimates of immigration rates and associated 95% credible intervals (CI, lower: 2.5%, upper: 97.5%) for Humboldt's flying squirrels (*Glaucomys oregonensis*; HFS) and Townsend's chipmunks (*Neotamias townsendii*; TC) captured 2011–2019 on the H. J. Andrews Experimental Forest in Oregon. Age and sex categories include juvenile (J), subadult (SA), adult (A), male (M), and female (F)

Species	Sex	Age	Year	Mean	95% CI	
					Lower	Upper
HFS	F	J	2012	0.21	0.17	0.26
			2013	0.06	0.05	0.08
			2014	0.05	0.04	0.06
			2015	0.06	0.05	0.08
			2016	0.05	0.04	0.07
			2017	0.18	0.15	0.22
			2018	0.09	0.07	0.11
			2019	0.03	0.02	0.04
		A	2012	0.19	0.15	0.23
			2013	0.05	0.04	0.07
			2014	0.04	0.03	0.05
			2015	0.05	0.04	0.07
			2016	0.04	0.03	0.06
			2017	0.16	0.13	0.20
			2018	0.08	0.06	0.09
			2019	0.02	0.01	0.04
	M	J	2012	0.26	0.21	0.31
			2013	0.07	0.06	0.09
			2014	0.06	0.05	0.08
			2015	0.07	0.06	0.09
			2016	0.06	0.04	0.08
			2017	0.23	0.19	0.27
			2018	0.11	0.08	0.13
			2019	0.03	0.02	0.05
		A	2012	0.23	0.19	0.28
			2013	0.06	0.05	0.08
			2014	0.05	0.04	0.07
			2015	0.06	0.05	0.08
			2016	0.05	0.04	0.07
			2017	0.20	0.16	0.24
			2018	0.09	0.07	0.12
			2019	0.03	0.02	0.05
TC	F	A	2012	0.22	0.19	0.24
			2013	0.36	0.33	0.40
			2014	0.17	0.15	0.19
			2015	0.15	0.13	0.17
			2016	0.23	0.20	0.26
			2017	0.25	0.22	0.28
			2018	0.21	0.18	0.23
			2019	0.35	0.30	0.42
	M		2012	0.30	0.27	0.34
			2013	0.51	0.46	0.56
			2014	0.24	0.21	0.26
			2015	0.21	0.18	0.24
			2016	0.32	0.28	0.36
			2017	0.35	0.31	0.39
			2018	0.29	0.25	0.32
			2019	0.49	0.42	0.58

**TABLE A4:** Age‐ and sex‐specific apparent survival emigration adjustment estimates and associated 95% credible intervals (CI, lower: 2.5%, upper: 97.5%) for Humboldt's flying squirrels (*Glaucomys oregonensis*; HFS) and Townsend's chipmunks (*Neotamias townsendii*; TC) captured 2011–2019 on the H. J. Andrews Experimental Forest in Oregon. Age and sex categories include juvenile (J), subadult (SA), adult (A), male (M), and female (F)

Species	Sex	Age	Interval	Mean	95% CI	
					Lower	Upper
HFS	F	J	2011–2012	0.17	0.10	0.23

2012–2013			0.03	0.02	0.05

2013–2014			0.03	0.02	0.04

2014–2015			0.03	0.02	0.05

2015–2016		0.02	0.01	0.03

2016–2017		0.14	0.08	0.20

2017–2018		0.04	0.01	0.08

2018–2019		0.01	0.00	0.02

SA		2011–2012	0.14	0.05	0.21

		2012–2013	0.05	0.03	0.06

		2013–2014	0.03	0.02	0.04

		2014–2015	0.02	0.01	0.04

2015–2016	0.03	0.02	0.05

2016–2017	0.11	0.06	0.17

2017–2018	0.06	0.04	0.08

2018–2019	0.02	0.00	0.03

A	2011–2012	0.14	0.05	0.20

	2012–2013	0.03	0.02	0.04

	2013–2014	0.03	0.02	0.04

	2014–2015	0.02	0.02	0.03

2015–2016	0.03	0.02	0.04

2016–2017	0.09	0.06	0.12

2017–2018	0.05	0.03	0.07

2018–2019	0.01	0.01	0.02

M	J	2011–2012	0.18	0.10	0.27

2012–2013		0.03	0.02	0.05

2013–2014		0.02	0.01	0.03

2014–2015	0.02	0.01	0.04

2015–2016	0.02	0.01	0.04

2016–2017	0.12	0.06	0.21

2017–2018	0.04	0.02	0.08

2018–2019	0.01	0.00	0.02

SA	2011–2012	0.18	0.11	0.25

	2012–2013	0.05	0.03	0.07

	2013–2014	0.03	0.02	0.04

2014–2015	0.03	0.02	0.05

2015–2016	0.02	0.01	0.04

2016–2017	0.16	0.10	0.22

2017–2018	0.06	0.04	0.09

2018–2019	0.01	0.00	0.03

A	2011–2012	0.17	0.06	0.25

	2012–2013	0.04	0.03	0.05

	2013–2014	0.03	0.02	0.04

2014–2015	0.03	0.02	0.05

2015–2016	0.02	0.01	0.03

2016–2017	0.15	0.10	0.21

2017–2018	0.05	0.03	0.07

2018–2019	0.02	0.01	0.03
TC	F	J	2011–2012	0.06	0.03	0.11
			2012–2013	0.14	0.05	0.29
			2013–2014	0.05	0.02	0.08
			2014–2015	0.03	0.01	0.07
			2015–2016	0.05	0.01	0.11
			2016–2017	0.07	0.03	0.13
			2017–2018	0.06	0.02	0.13
			2018–2019	0.08	0.02	0.18
		A	2011–2012	0.10	0.08	0.13
			2012–2013	0.21	0.16	0.27
			2013–2014	0.05	0.04	0.06
			2014–2015	0.07	0.05	0.08
			2015–2016	0.11	0.08	0.15
			2016–2017	0.08	0.06	0.11
			2017–2018	0.06	0.04	0.08
			2018–2019	0.05	0.01	0.11
	M	J	2011–2012	0.09	0.04	0.15
			2012–2013	0.25	0.10	0.47
			2013–2014	0.08	0.04	0.12
			2014–2015	0.07	0.03	0.12
			2015–2016	0.14	0.06	0.26
			2016–2017	0.06	0.02	0.13
			2017–2018	0.07	0.02	0.18
			2018–2019	0.12	0.02	0.27
		A	2011–2012	0.10	0.07	0.14
			2012–2013	0.33	0.24	0.43
			2013–2014	0.10	0.08	0.12
			2014–2015	0.08	0.06	0.10
			2015–2016	0.12	0.09	0.15
			2016–2017	0.13	0.09	0.18
			2017–2018	0.08	0.05	0.11
			2018–2019	0.07	0.02	0.16

**TABLE A5:** Ratios of adult to juvenile survival, including apparent annual survival (*φ*) and adjusted annual survival (*φ*_adjusted_), for male (M) and female (F) Humboldt's flying squirrels (*Glaucomys oregonensis*; HFS) and Townsend's chipmunks (*Neotamias townsendii*; TC) captured during 2011–2019 on the H. J. Andrews Experimental Forest in Oregon

Species	Estimate	Sex	Mean	Min	Max
HFS	*φ*	F	1.22	0.74	2.02
		M	1.49	1.24	1.78
	*φ*_adjusted_	F	1.19	0.72	2.01
		M	1.46	1.22	1.78
TC	*φ*	F	1.40	0.62	2.18
		M	1.18	0.61	2.02
	*φ*_adjusted_	F	1.40	0.62	2.18
		M	1.18	0.61	2.03
